# Non-Expanding Plaster

**Published:** 1904-11

**Authors:** 


Non-Expanding Plaster.—The author reports the results of his experiments with plaster of Paris, begun in view of devising means by which to prevent, if possible, the expansion of plaster during the setting process. His test consisted mainly in the mixing of plaster with a saturated solution of calcium oxid in water previously filtered, boiled, and cooled; with lime-water prepared without filtering or boiling; with a solution of gum arabic; and with lime-water to which a little salt had been added. A test was also made with plain boiled water, allowed to cool. Through these various experiments the author found that plaster mixed with a saturated solution of calcium oxid in water filtered, boiled, and cooled does not expand. The gum arabic, solution likewise prevents expansion, but the resulting plaster is less dense than when mixed with lime-water.—*International.*

